# N6-methylandenosine-related immune genes correlate with prognosis and immune landscapes in gastric cancer

**DOI:** 10.3389/fonc.2022.1009881

**Published:** 2022-11-29

**Authors:** Yuancheng Huang, Yushan Zou, Yanhua Tian, Zehong Yang, Zhengkun Hou, Peiwu Li, Fengbin Liu, Jiasheng Ling, Yi Wen

**Affiliations:** ^1^ First Clinical Medical College, Guangzhou University of Chinese Medicine, Guangzhou, Guangdong, China; ^2^ Department of Gastroenterology, The First Affiliated Hospital of Guangzhou University of Chinese Medicine, Guangzhou, Guangdong, China; ^3^ Department of Gastroenterology, Baiyun Branch of the First Affiliated Hospital of Guangzhou University of Chinese Medicine, Guangzhou, Guangdong, China; ^4^ Department of Gastroenterology, Huizhou Hospital of Traditional Chinese Medicine, Huizhou, Guangdong, China

**Keywords:** gastric cancer, N6-methyladenosine, immune genes, prognostic signature, tumor microenvironment

## Abstract

**Objectives:**

This study aimed to probe into the significance of N6-methyladenosine (m^6^A)-related immune genes (m^6^AIGs) in predicting prognoses and immune landscapes of patients with gastric cancer (GC).

**Methods:**

The clinical data and transcriptomic matrix of GC patients were acquired from The Cancer Genome Atlas database. The clinically meaningful m^6^AIGs were acquired by univariate Cox regression analysis. GC patients were stratified into different clusters *via* consensus clustering analysis and different risk subgroups *via* m^6^AIGs prognostic signature. The clinicopathological features and tumor microenvironment (TME) in the different clusters and different risk subgroups were explored. The predictive performance was evaluated using the KM method, ROC curves, and univariate and multivariate regression analyses. Moreover, we fabricated a nomogram based on risk scores and clinical risk characteristics. Biological functional analysis was performed based on Gene Ontology and Kyoto Encyclopedia of Genes and Genomes pathways. The connectivity map was used to screen out potential small molecule drugs for GC patients.

**Results:**

A total of 14 prognostic m^6^AIGs and two clusters based on 14 prognostic m^6^AIGs were identified. A prognostic signature based on 4 m^6^AIGs and a nomogram based on independent prognostic factors was constructed and validated. Different clusters and different risk subgroups were significantly correlated with TME scores, the distribution of immune cells, and the expression of immune checkpoint genes. Some malignant and immune biological processes and pathways were correlated with the patients with poor prognosis. Ten small molecular drugs with potential therapeutic effect were screened out.

**Conclusions:**

This study revealed the prognostic role and significant values of m^6^AIGs in GC, which enhanced the understanding of m^6^AIGs and paved the way for developing predictive biomarkers and therapeutic targets for GC.

## Introduction

As the fifth most prevalent cancer and third most lethal neoplasm worldwide, gastric cancer (GC) presents a nonnegligible threat to global health (1). Key contributors to the high mortality of GC include its low early diagnostic rate and poor curative effect in the advanced stage (2, 3). Recently, targeted therapy and immunotherapy have shown their clinical efficacy in improving the prognosis of GC patients (4). Therefore, searching for novel biomarkers and effective therapeutic targets for GC patients is of increasing importance.

RNAs in eukaryotic cells are discovered to have over 100 different kinds of post-transcriptional modifications, which are associated with RNA stability, processing, and so on (5). N6-methyladenosine (m^6^A), is a methylation modification of the sixth nitrogen atom of adenine, widely present in messenger RNAs, pri-microRNAs, long non-coding RNAs, and circular RNAs, is the most dominant and enriched kind of internal RNA modification in eukaryotes (6). Involving multiple tumor-related biological processes, m^6^A modification is a reversible process, which is controlled under the three types of m^6^A regulator (methyltransferases “writers”, for demethylases “erasers”, and binding proteins “readers”) (7), which involved in various biological processes related to the occurrence and progression of different cancers, including GC (8, 9). For example, METTL3 could improve the stability of HDGF by stimulating m^6^A modification, which accelerates the progression of GC (10). Moreover, METTL3, through an m^6^A DGCR8-dependent method, could accelerate the processing of pri-miR-17, which facilitates the development of GC by activating the AKT/mTOR pathway (11). The m^6^A modification of METTL3 also enhances the expression of long non-coding RNAs THAP7-AS1, which exerts oncogenic functions in GC (12).

There is plenty of evidence that the immune system is pivotal to the occurrence and development of cancers (13). Immune cells take key a part in immune surveillance, which recognizes cancer-associated antigens and eradicates cancer cells (14). In recent years, accumulating evidence has shown that cancer immunotherapy has significant efficacy, which could prolong the overall survival (OS) of patients (15). However, the number of patients achieving clinical benefits is small, primarily due to the immunosuppression in the complicated tumor microenvironment (TME) (16). The TME, composing immune-related cells, blood vessels, cytokines, extracellular matrix, adipocytes, myofibroblasts, fibroblasts, and neuroendocrine cells, induces changes in the phenotype of cancer cells and immune cells to promote immune escape by complex molecular mechanisms (17, 18). The association of immune cells and immune-related genes with GC have been demonstrated by an increasing number of studies (19, 20). Additionally, immune-related genes and immune cells also present tremendous latent value in serving as novel prognostic biomarkers of GC patients (21–23).

Epigenetic and immune therapy has been under concentrated investigation for many years, and there has been evidence that the inhibitors of histone methyltransferase and DNA methyltransferase could facilitate anti-tumor immunity (24). Recently, the RNA modification m^6^A has been identified as a key regulator in the immune system, such as immune recognition and immune responses (25, 26). For instance, the m^6^A modification could regulate durable neoantigen-specific immunity (27). The m^6^A modification of CD40 and CD80 could stimulate T-cell activation by enhancing their translation in dendritic cells (28). Notwithstanding, the full role of m^6^A-related immune genes (m^6^AIGs) for patients with GC has never been systematically evaluated.

Here, we analyzed The Cancer Genome Atlas (TCGA) database for m^6^AIGs involved in GC, identified two clusters based on prognostic m^6^AIGs, and constructed an m^6^AIGs prognostic signature (m^6^AIGs-PS) for GC patients. Then, we further explore the correlation of m^6^AIGs with clinicopathological features, TME scores, TME infiltrating immune cells (TIICs), and immune checkpoint genes (ICGs), as well as biological function. Subsequently, based on the connectivity map (CMap) database, small molecule drugs having the potential to suppress high-risk gene expression in GC were identified. These findings as a revelation of the critical role of m^6^AIGs and disclosed the latent relationship and the underlying mechanism between m^6^AIGs and tumor-immune interactions.

## Methods and materials

### Data collection

Raw data, composed of transcriptomic matrix and clinical information of GC (375 samples) and normal tissues (32 samples), was downloaded from the TCGA database (https://portal.gdc.cancer.gov/).

### Selection of immune-related gene and m^6^A-related regulators

The immune-related genes were downloaded from InnateDB (https://www.innatedb.com/) and ImmPort (https://www.immport.org/) respectively (29, 30). The above-obtained genes were combined and a total of 3179 immune-related genes were included. Based on published data (6, 7, 31), 23 m^6^A-related regulators, including ZC3H13, RBM15, RBM15B, METTL3, METTL16, METTL14, YTHDC1, YTHDC2, WTAP, VIRMA, HNRNPC, FMR1, LRPPRC, YTHDF1, YTHDF2, YTHDF3, HNRNPA2B1, IGFBP3, FTO, IGFBP2, RBMX, IGFBP1, and ALKBH5, were used in our study.

### Identification of prognostic m^6^AIGs

Primarily, m^6^AIGs were filtrated by performing correlation analysis between m^6^A-related regulators and immune-related genes. To filtrate the prognostic m^6^AIGs, a univariate Cox regression analysis was conducted. The correlation analysis between prognostic m^6^AIGs and m^6^A-related regulators was implemented.

### Consistent clustering of m^6^AIGs

To determine subgroups of GC, we used the R package “ConsensusClusterPlus” for the consistent clustering based on the expression pattern of prognostic m^6^AIGs as the previous study (32). Using the K-mean cluster algorithm and the Euclidean squared distance metric, the optimal clustering amount was confirmed by the clustering score.

To explore the role of m^6^AIGs in GC, the different clinicopathological features, OS, TME scores, the content of TIICs, and the expression of ICGs of different clusters were compared as the previous study (33). The content of TIICs was recognized through CIBERSORT (34), and TME immune scores were counted based on the “ESTIMATE” package (35).

### Establishment and validation of m^6^AIGs-PS

The included patients were randomly segmented into two cohorts of approximately equal numbers (the training cohort with 169 samples and the test cohort containing 168 samples). Thereafter, based on prognostic m^6^AIGs and the training cohort, the least absolute shrinkage and selection operator (LASSO) Cox regression algorithm was performed to establish m^6^AIGs-PS, which could screen out m^6^AIGs with optimal performance and calculate their coefficients. The risk score (RS) was estimated using the following formula: RS= coef (m^6^AIGs1) × expr (m^6^AIGs1) + coef (m^6^AIGs2) × expr (m^6^AIGs2) +…… + coef (m^6^AIGsn) × expr (m^6^AIGsn). The coef (m^6^AIGsn) was the coefficient of prognostic m^6^AIGs, and expr (m^6^AIGsn) was the expression of m^6^AIGs. Based on m^6^AIGs-PS, the training cohort was segmented into two groups: the high-risk group and the low-risk group. The predictive performance of m^6^AIGs-PS was verified by the receiver operating characteristic (ROC) curves and Kaplan-Meier (KM) analysis. Moreover, to further validate the accuracy of m^6^AIGs-PS, the same methods were performed in the test cohort and the combined cohort. Furthermore, principal component analysis (PCA) was used to assess the clustering ability of RS. In addition, univariate and multivariate Cox regression analyses were used to examine the predictive performance of RS.

### Establishment and evaluation of a nomogram

To fully expand the predictive performance of m^6^AIGs-PS, we constructed another quantitative method, nomogram to predict the individual probability of survival based on the independent prognostic factors by using “rms” package in R (36).

### Clinical application of m^6^AIGs-PS

To explore the clinical application of m^6^AIGs-PS, the different clinicopathological characteristics, the expression of ICGs, the content of TIICs, and the TME scores of different risk subgroups were compared. Additionally, the association between OS and the content of TIICs and TME scores was also investigated.

### Functional enrichment analysis

To explore the biological functions associated with m^6^AIGs, Gene Set Enrichment Analysis (GSEA), Kyoto Encyclopedia of Genes and Genomes (KEGG) pathway, and Gene Ontology (GO) analysis were performed. Genes in different risk subgroups and different clusters were functionally annotated by GSEA. With the “edgeR” package in R, differentially expressed genes (DEGs) (|fold change| > 1 and *p* < 0.05) between different risk subgroups and different clusters were screened out and applied to GO and KEGG pathway analysis.

### Screening of potential small molecule drugs

To predict potential small molecule drugs for GC, the connectivity map (CMap) (https://portals.broadinstitute.org/cmap/) database was used (37). These small molecule drugs were identified based on DEGs between different risk subgroups. The drugs (*p* < 0.05) were ranked according to negative connectivity value. The 3D structures of the three most significant drugs were obtained from PubChem (https://pubchem.ncbi.nlm.nih.gov/).

### Quantitative reverse transcription-polymerase chain reaction

The RNA Purification Kit (EZBioscience, USA), Color Reverse Transcription Kit (EZBioscience, USA), and Color SYBR Green qPCR Master Mix (EZBioscience, USA) were used for RNA extraction, reverse transcription, and qRT-PCR according to their manufacturer’s instructions. GAPDH was used as an endogenous control. Primers sequences used in our study were as follows: GAPDH forward 5’-GGACCTGACCTGCCGTCTAG-3’, and reverse 5’-GTAGCCCAGGATGCCCTTGA-3’; PAEP forward 5’-GCGACCAACAACATCTCCCTC-3’, and reverse 5’-CGACACAACTCTTCTTCCAGC-3’; NPR1 forward 5’-GCAAAGGCCGAGTTATCTACATC-3’, and reverse 5’-AACGTAGTCCTCCCCACACAA-3’; GLP2R forward 5’-CTTATTCCTTTCCTGGC-3’, and reverse 5’-GACAGGTAGGACATCCACC-3’; FANCC forward 5’-GGCAAAAGCTTGTTGGAATC-3’, and reverse 5’-CCAGGAGTTAAGTTTTGATTGTCC-3’.

### Statistical analysis

The expression data were compared using a one-way analysis of variance; the clinicopathological characteristics of different subgroups were compared using the chi-square test; the KM method was used to perform a bilateral logarithmic rank test in OS analysis; *p* < 0.05 were regarded as statistically significant. R v4.0.3 (https://www.r-project.org/) or Spss software (Version 20.0) or GraphPad Prism software (Version 8.0) were used for all statistical analyses.

## Results

### Identification of prognostic m^6^AIGs in GC

**Figure 1:**
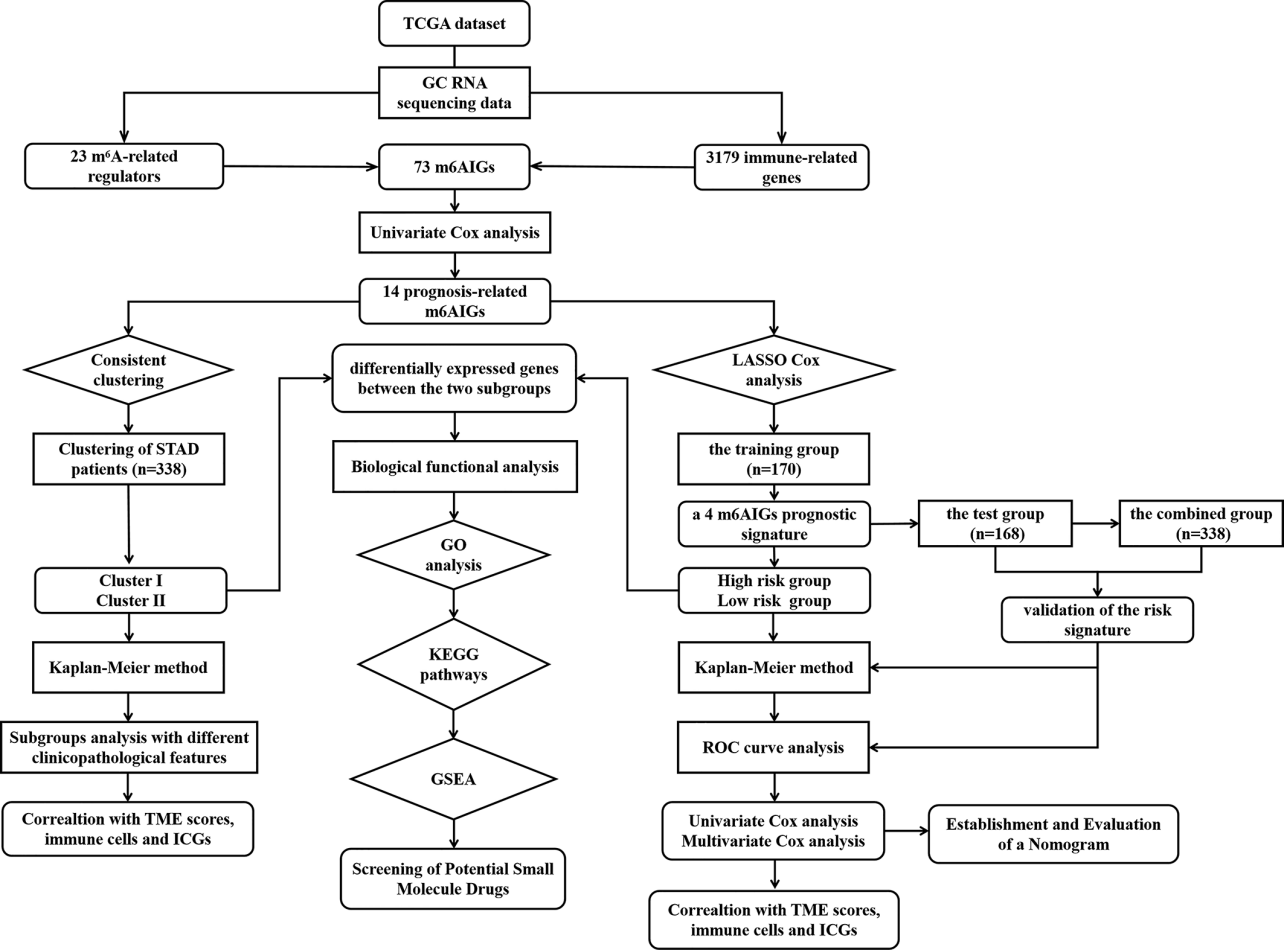
The flow chart of the study.

**Figure 2:**
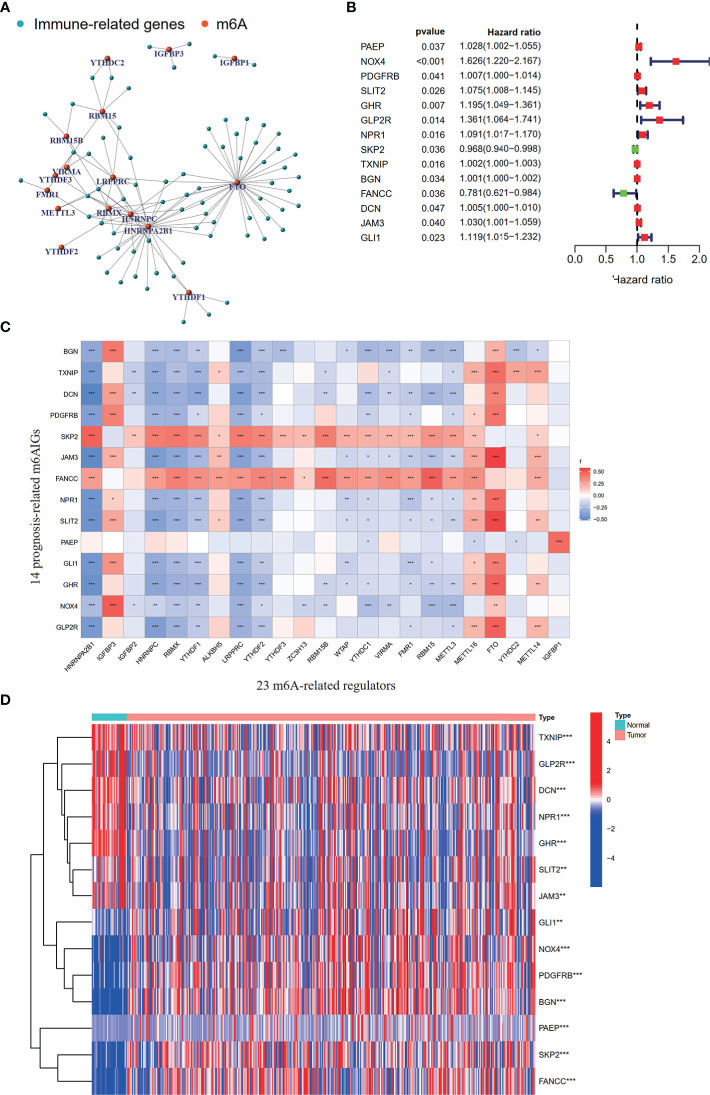
Processing of data and acquisition of prognosis-related m^6^AIGs. **(A)** The network of the 73 m^6^AIGs and 23 m^6^A-related regulators. **(B)** The hazard ratio 95% confidence interval of 14 prognostic m^6^AIGs were estimated by univariate Cox regression. **(C)** Pearson correlation analysis between 23 m^6^A-related regulators and 14 prognostic m^6^AIGs. **(D)** The heatmap with differential expression of 14 prognostic m^6^AIGs between the tumor group and the normal group.*p <0.05, **p <0.01, ***p <0.001.

To clearly illustrate the process of our study, a flow chart is shown in **Figure 1**. The expression levels of m^6^A-related regulators and immune-related genes were extracted respectively. 73 m^6^AIGs were identified (|cor| > 0.4, *p* < 0.001) through coexpression analysis. 23 m^6^A-related regulators and 73 m^6^AIGs were shown in co-expression network (**Figure 2A**). 14 m^6^AIGs having a dramatic correlation with OS were identified *via* univariate Cox analyses (*p* < 0.05) (**Figure 2B**). The co-expression relationship between the candidate m^6^AIGs and m^6^A-related regulators was shown in **Figure 2C**. The expression pattern of the 14 prognosis-related m^6^AIGs was shown in the heatmap (**Figure 2D**).

### Consensus clustering of prognosis-related m^6^AIGs identified two GC clusters with different clinicopathological characteristics and immune landscape

**Figure 3:**
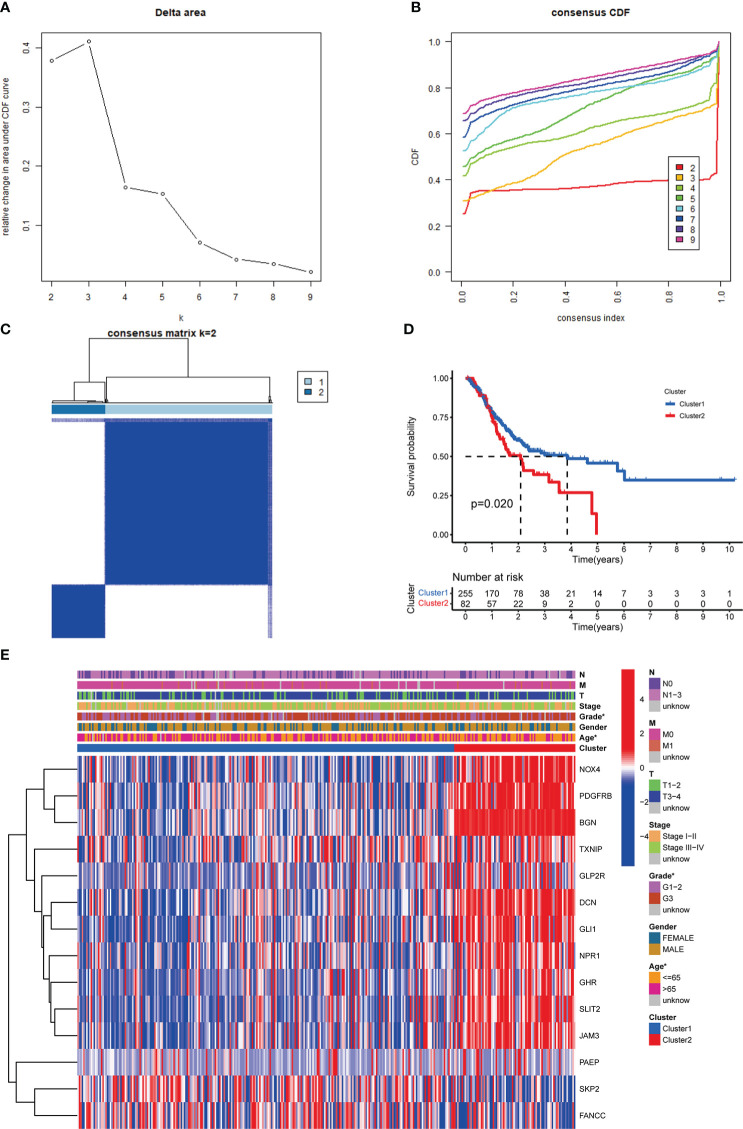
Identification of m^6^AIGs-based clusters of GC samples. **(A)** The consensus among clusters from 2 to 10 of (k). **(B)** Delta area curve of consensus clustering. **(C)** Consensus clustering of GC samples with k = 2. **(D)** KM analyses for GC patients in clusters I and II. **(E)** The distribution of clinicopathological characteristics and the expression of 14 prognostic m^6^AIGs in clusters I and II. *p < 0.05.

Consistent clustering analysis of GC patients was performed and we found that the optimal cluster number was two (**Figures 3A-C**). We found significant differences in OS between clusters I and II *via* KM analysis (**Figure 3D**). The relationship between clinicopathological characteristics and different clusters was shown in **Figure 3E**.

**Figure 4:**
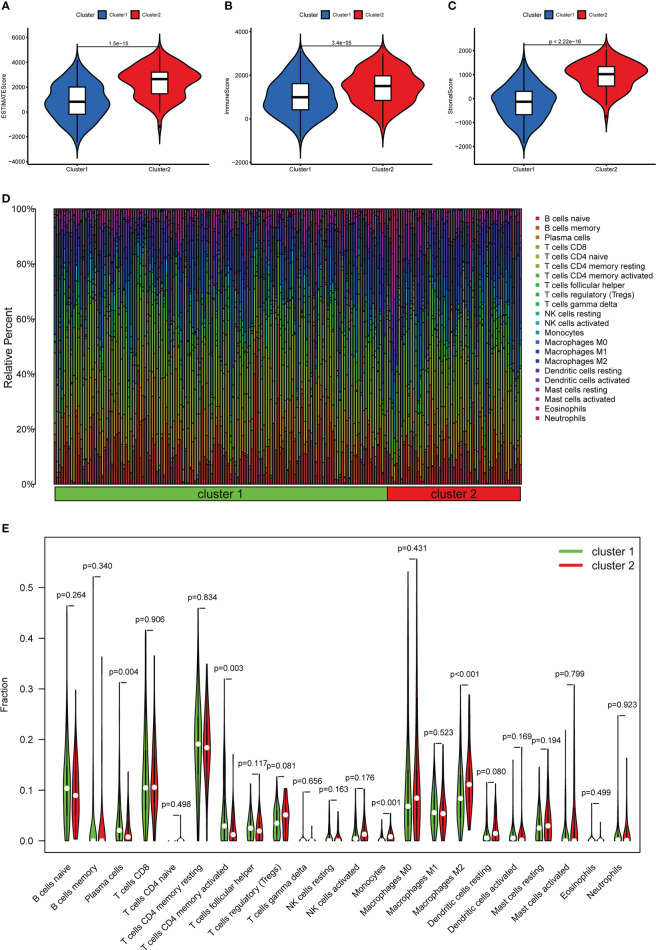
The relationship between different clusters and TME. **(A–C)** Comparison of ESTIMATE scores, Immune scores, and Stromal scores in clusters I and II. **(D)** The distribution of TIICs in clusters I and II. **(E)** Violin plot showing differences in TIICs between clusters I and II.

In terms of TME scores, the ESTIMATE, stromal, and immune scores in cluster II were significantly higher than those in cluster I (**Figures 4A-C**). Furthermore, in terms of the content of TIICs, cluster I contained more CD4 memory activated T cells (*p* = 0.003) and plasma cells (*p* = 0.004), and cluster II had more monocytes (*p* < 0.001) and M2 macrophages (*p* < 0.001) (**Figures 4D, E**).

**Figure 5:**
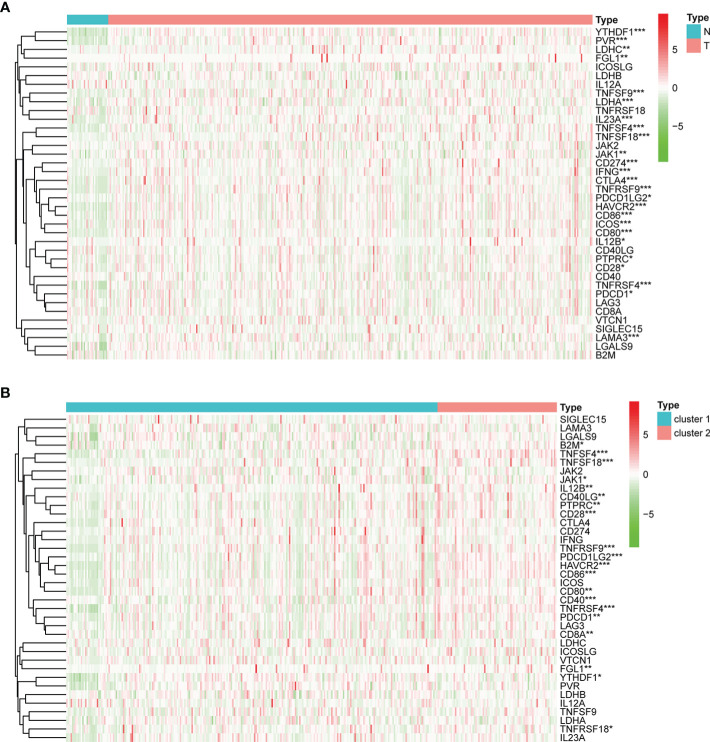
The expression of ICGs in the different subgroups. **(A)** Heatmap for the differential expression of ICGs between normal and tumor tissues. **(B)** Heatmap for the differential expression of ICGs between clusters I and II. *p < 0.05, **p < 0.01, and ***p < 0.001.

In addition, we also investigated the expression levels of 38 ICGs obtained from previous studies (27, 38–41) in different clusters. Firstly, compared with the adjacent tissues, 25 ICGs were differentially expressed in the gastric tumor tissues (*p* < 0.05) (**Figure 5A**). Then, we observed that 20 ICGs were differentially expressed between the two clusters (*p* < 0.05) (**Figure 5B**). To be more specific, TNFSF4, CD86, PDCD1LG2, TNFRSF4, HAVCR2, CD28, TNFSF18, TNFRSF9, PTPRC, PDCD1, IL12B, CD80, JAK1were significantly highly expressed in gastric tumor tissues and cluster II that associated with worse OS.

### Construction and validation of m^6^AIGs-PS

**Figure 6:**
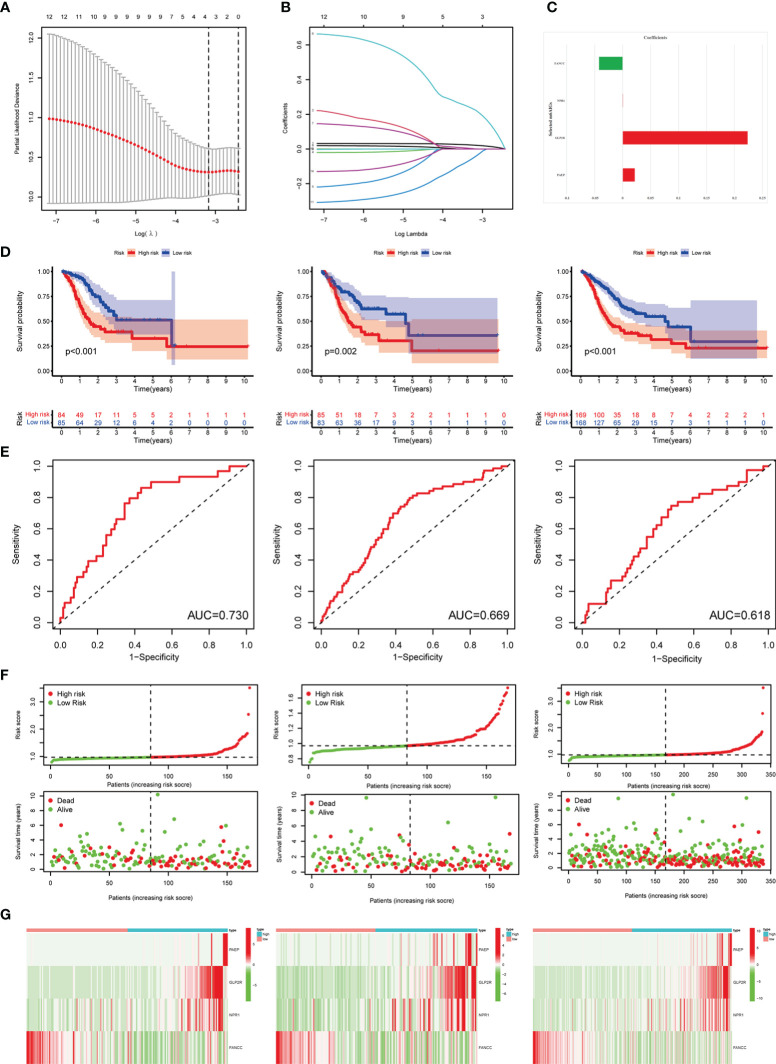
Construction and validation of the m^6^AIGs-PS. **(A)** The number that corresponded to the point with the smallest cross-verification error was the gene numbers included in the LASSO regression risk model. **(B)** The lines of different colors represent the trajectory of the correlation coefficient of different factors in the model with the increase of Log Lamda. **(C)** LASSO coefficients of 4 selected prognostic m^6^AIGs. **(D)** KM analysis of patients in the low- and high-risk groups in the training cohort, the test cohort, and the combined cohort. **(E)** ROC analysis for OS prediction in the training cohort, the test cohort, and the combined cohort. **(F)** The distribution plots of RS and survival status in the training group, the test group, and the combined group. **(G)** Heatmap of four-gene profiles in the low- and high-risk groups in the training cohort, the test cohort, and the combined cohort.

**Figure 7:**
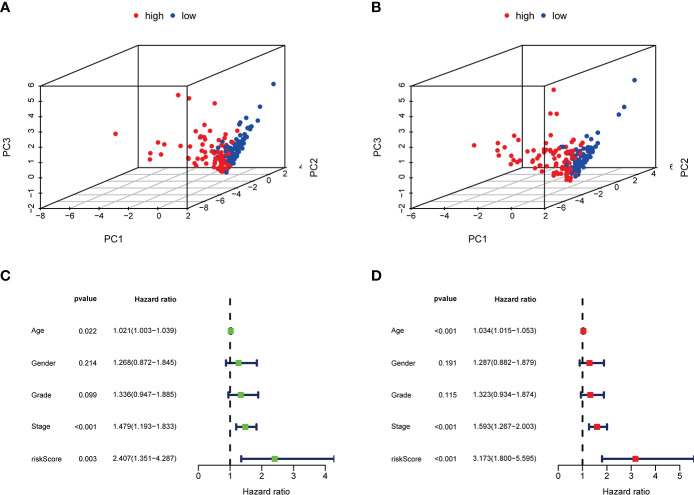
PCA analysis, univariate and multivariate Cox regression analyses of m^6^AIGs-PS. **(A, B)** PCA analysis in the training group and the test group. **(C)** Univariate Cox regression analyses in the combined group. **(D)** Multivariate Cox regression analyses in the combined group.

After using the LASSO method in the training cohort (n = 169), 4 m^6^AIGs were chosen to establish m^6^AIGs-PS (**Figures 6A-C**). Using the median RS as a cutoff value, the training cohort was segmented into the low-risk and high-risk groups. KM survival analysis proved that the high-risk group had a significantly shorter survival time than the low-risk group (*p* < 0.01) (**Figure 6D**). The value of the area under the curve in the time-dependent ROC curve was 0.730 (**Figure 6E**), suggesting the powerful prediction performance of m^6^AIGs-PS in the training cohort. Furthermore, the distribution plot of RS and survival status demonstrated that the higher RS, the more deaths of patients (**Figure 6F**). To further validate the m^6^AIGs-PS, verification analysis in the test group (n = 168) and the combined group (n = 337) was performed. Consequently, the m^6^AIGs-PS also had well-prediction performances in these two cohorts. The expression pattern of selected m^6^AIGs of the different cohorts in different risk subgroups was shown in **Figure 6G**. Additionally, PCA analysis demonstrated a reliable clustering ability of RS (**Figures 7A, B**). Moreover, univariate Cox regression and multivariable Cox regression analyses demonstrated RS was significantly correlated with OS in addition to age and stage (*p* < 0.05) (**Figures 7C, D**).

### Establishment and evaluation of a nomogram based on independent prognostic factors

**Figure 8:**
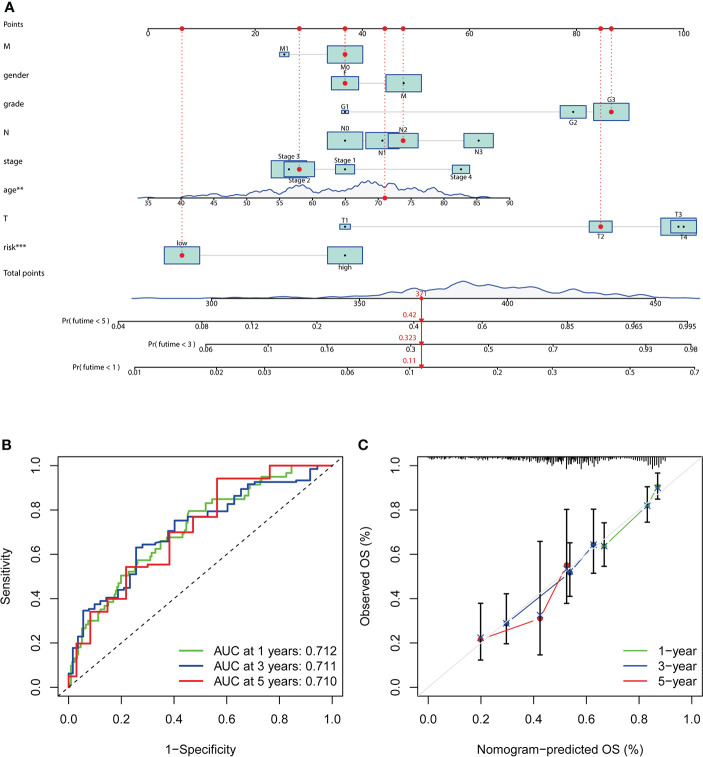
Establishment and evaluation of a nomogram. **(A)** Nomogram based on RS, age at diagnosis, gender, grade, clinical stage, and stage TMN. **(B)** The ROC analyses of the nomogram in predicting 1-, 3-, and 5-year OS. **(C)** The calibration curves show the concordance between the actual and nomogram-predicted probability of 1-, 3-, and 5-year OS.

The nomogram comprising clinical risk features and RS was fabricated (**Figure 8A**), which showed robust accuracy in predicting 1-, 3-, and 5-year OS (AUC = 0.712, 0.711, and 0.710, respectively) (**Figure 8B**). Moreover, the calibration curves demonstrated a good match between the actual and nomogram-predicted probability (**Figure 8C**). These data indicated that it had the latent force to be used as a quantitative instrument to predict the OS for patients with GC, which had particular importance in clinical practice.

### Subgroup analysis with different clinicopathological features

**Figure 9:**
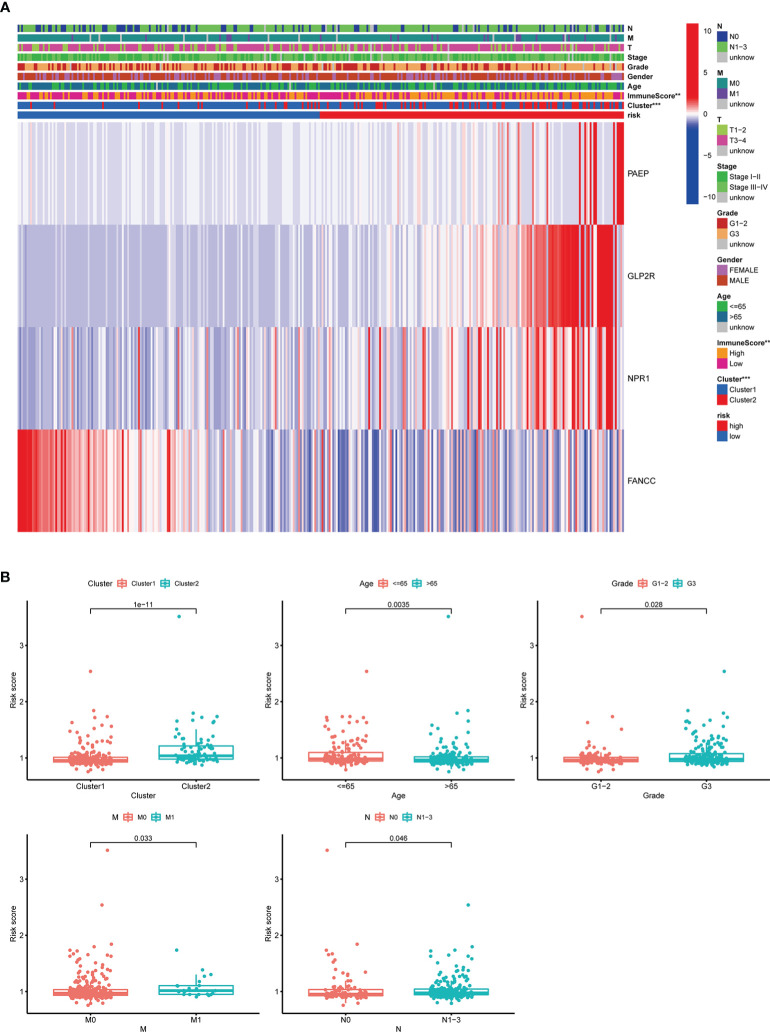
The relationship between RS and clinicopathological characteristics, and TME. **(A)** The heatmap showed the expression of 4 prognostic m^6^AIGs, the distribution of clinicopathological characteristics, and the immune scores of TME in different risk subgroups. **p <0.01, ***p <0.001. **(B)** Significant differences in RS were observed between different clusters, age, stage M, stage N, and different tumor grades.

The distribution of clinicopathological features, the immune scores of TME, and the expression of 4 selected m^6^AIGs of patients in different risk subgroups were shown as a heatmap (**Figure 9A**). Significant differences were observed between the two subgroups according to cluster (*p* < 0.001) and the immune scores of TME (*p* < 0.001). Significant differences in RS were observed between: 1) different clusters (*p* < 0.001); 2) age (*p* < 0.01); 3) clinical stage (*p* < 0.05); 4) stage M (*p* < 0.01); 5) stage N (*p* < 0.05) (**Figure 9B**).

**Figure 10:**
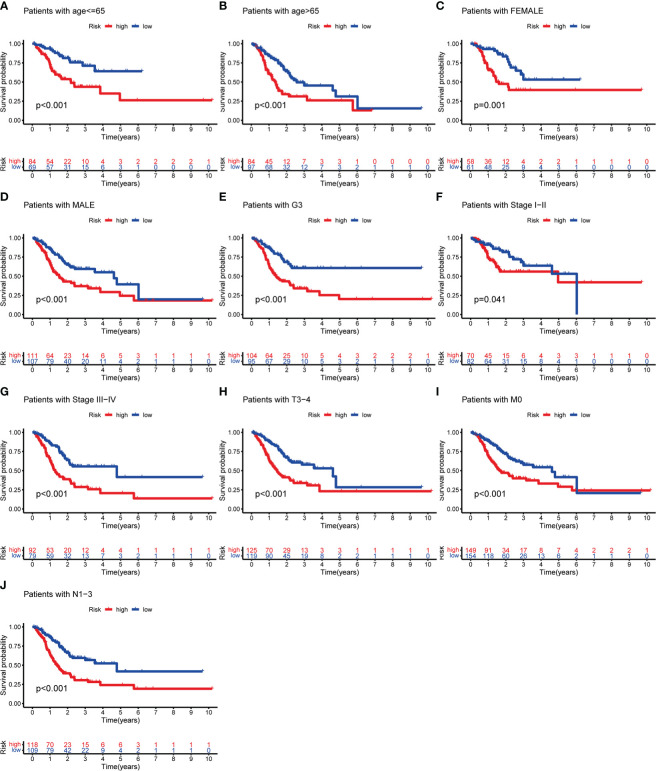
KM analysis with different clinicopathological features in different risk subgroups. **(A)** Age ≤ 65. **(B)** Age > 65. **(C)** Female. **(D)** Male. **(E)** G3. **(F)** Stage I-II. **(G)** Stage III-IV. **(H)** Stage T3-4. **(I)** Stage M0. **(J)** Stage N1-3.

To evaluate whether m^6^AIGs-PS could be a prognostic instrument for patients with diverse clinical characteristics, we stratified subgroups by age, gender, grade, clinical stage, stage T, stage M, and stage N. Consequently, the OS of the low-risk patients according to age(*p*< 0.001 in age ≤ 65 and age > 65), sex (*p* < 0.001 in female and male), grade (*p* < 0.001 in G3), stage I-II (*p* < 0.05), stage III-IV (*p* < 0.001), stage T3-4 (*p* < 0.001), stage M0 (*p* < 0.001) and stage N1-3 (*p* < 0.001) was significantly higher than those of the high-risk patients (**Figure 10**).

### Subgroup analysis with different immune landscape

**Figure 11:**
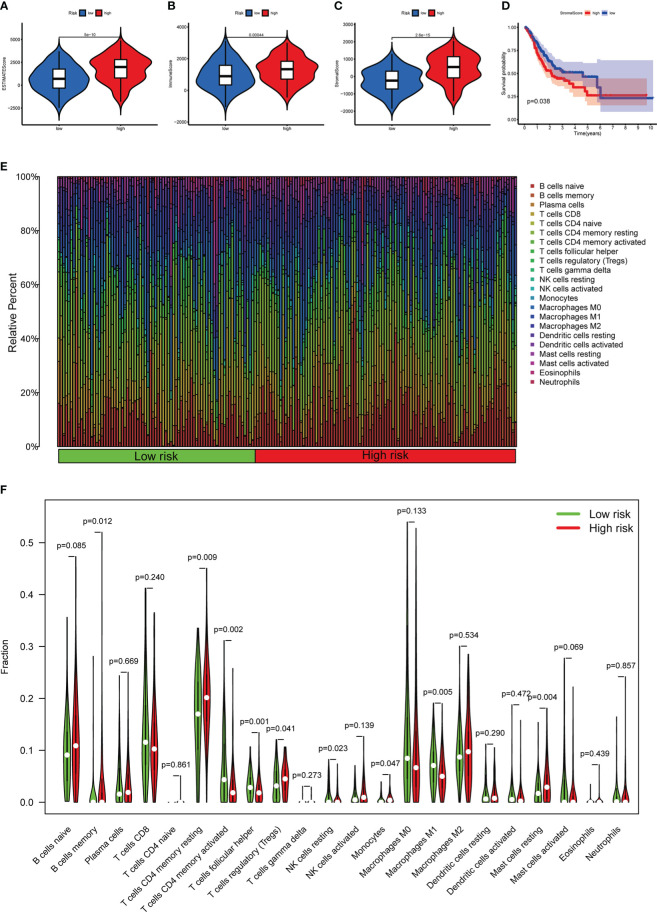
The relationship between different risk subgroups and TME. **(A–C)** Comparison of ESTIMATE scores, Immune scores, and Stromal scores in the high-risk group and low-risk group. **(D)** The OS of patients with high stromal scores was significantly lower than those with low stromal scores. **(E)** The distribution of TIICs in the high-risk group and low-risk group. **(F)** Violin plot showing differences in TIICs between the high-risk group and low-risk group.

**Figure 12:**
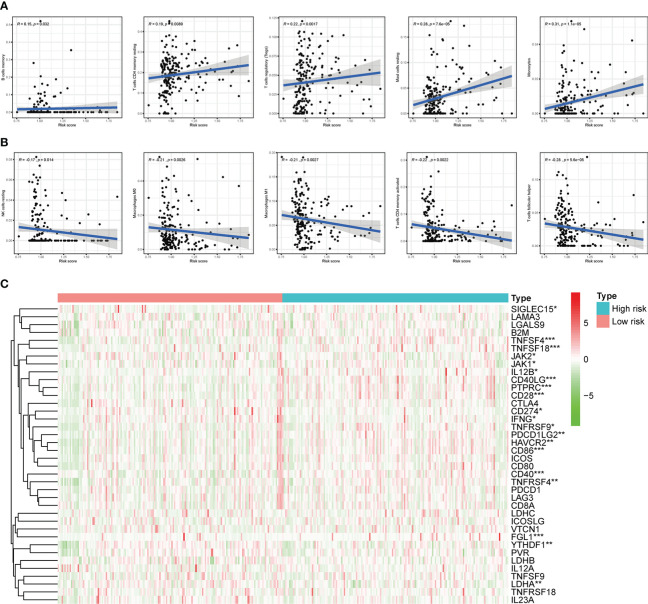
Correlation of subgroup with TME and ICGs. **(A)** The high-risk group has significant positive correlations with memory B cells, memory resting CD4 T cells, regulatory T cells, resting mast cells, and monocytes. **(B)** The high-risk group has significant negative correlations with resting NK cells, M0 Macrophages, M1 Macrophages, memory activated CD4 T cells, and follicular helper T cells. **(C)** The expression of ICGs between different risk subgroups. *p < 0.05, **p < 0.01, and ***p < 0.001.

Based on the previously mentioned currently accepted methods, the relationship between different subgroups and the immune landscape, including TME scores, TIICs and ICGs, was explored. In terms of TME scores, the ESTIMATE, stromal, and immune scores of the high-risk groups were significantly higher than those in the low-risk groups (**Figures 11A-C**). Moreover, we further found that the higher the stromal scores, the lower OS of patients (*p* < 0.05) (**Figure 11D**). As for TIICs, the correlation between the content of TIICs and subgroups was shown in **Figure 11E, F**. To be more specific, the RS had significant positive correlations with infiltrating levels of memory B cells (r = 0.15, *p* = 0.032), memory resting CD4 T cells (r = 0.19, *p* = 0.0089), regulatory T cells (r = 0.22, *p* = 0.0017), resting mast cells (r = 0.28, *p* = 7.6E-05), monocytes (r = 0.31, *p* = 1.1E-05) (**Figure 12A**), and had significant negative correlations with infiltrating levels of resting NK cells (r = -0.17, *p* = 0.014), M0 Macrophages (r = -0.21, *p* = 0.0026), M1 Macrophages (r = -0.21, *p* = 0.0027), memory activated CD4 T cells (r = -0.22, *p* = 0.0022), follicular helper T cells (r = -0.28, *p* = 5.6E-05) (**Figure 12B**). Concerning ICGs, in the high-risk group, seven ICGs (namely, TNFSF4, TNFSF18, CD40LG, PTPRC, CD28, CD86, CD40) were significantly highly expressed, while in the low-risk group, one ICGs (namely, FGL1) was significantly highly expressed (**Figure 12C**).

### Functional enrichment analysis

**Figure 13:**
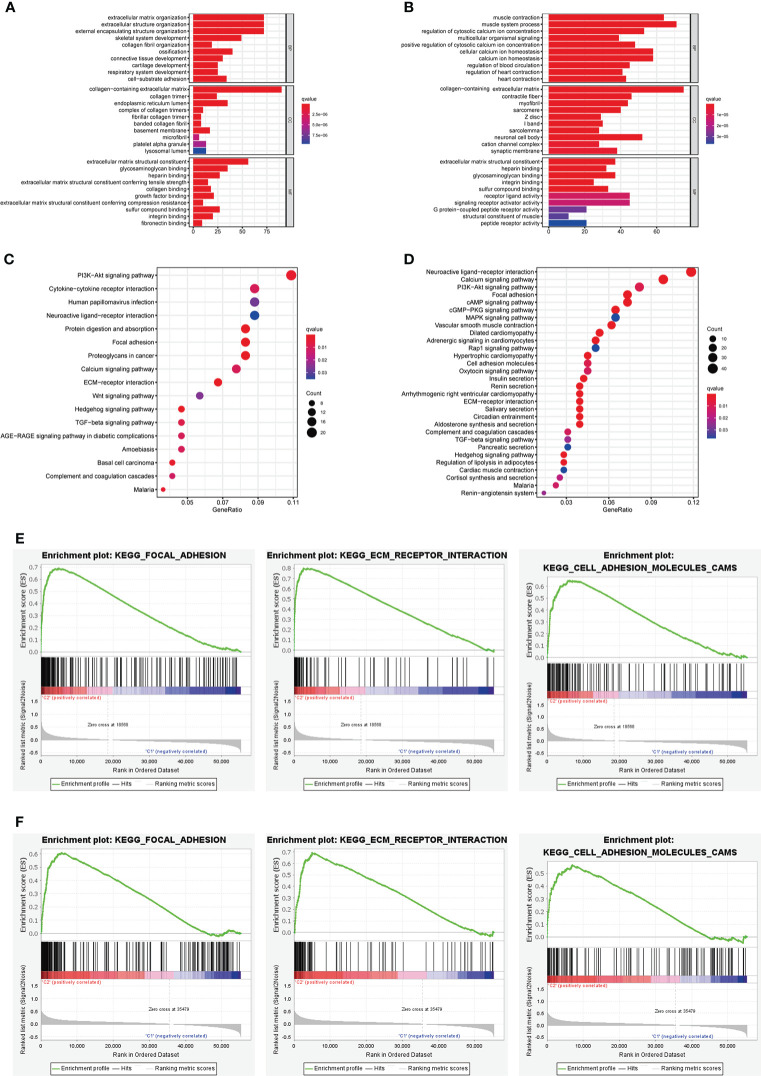
Biological functional analysis. **(A, B)** The bar plot of GO analysis in DEGs between different clusters and different risk subgroups. **(C, D)** The bubble plot of KEGG pathways analysis in DEGs between different clusters and different risk subgroups. **(E, F)** GSEA analysis for cluster II and high-risk group.

The results of GO analysis suggested that the DEGs in different risk subgroups and clusters were mainly enriched in extracellular structure-related biological processes, such as “extracellular matrix organization”, “collagen-containing extracellular matrix”, and “extracellular matrix structural constituent” (**Figures 13A, B**). KEGG pathway analysis results revealed that the DEGs in different clusters or risk groups were particularly enriched in malignancy-associated pathways, including “PI3K-Akt signaling pathway”, “Focal adhesion,” and “Wnt signaling pathway”. (**Figures 13C, D**). Moreover, the result of GSEA showed that some cancer-related pathways, such as “Focal adhesion”, “ECM−receptor interaction”, and “Cell adhesion molecules”, were highly active in cluster II and high-risk group (**Figure 13E, F**).

### CMap database analysis

**Figure 14:**
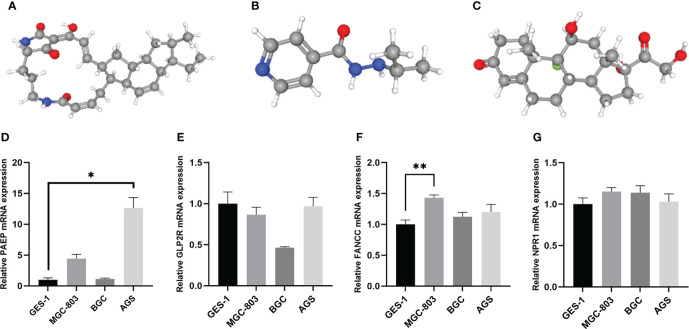
The 3D structure of the three small molecule drugs for GC and validation of the expression levels of the prognostic m^6^AIGs in cell lines. **(A)** Ikarugamycin. **(B)** Iproniazid. **(C)** Fludrocortisone. **(D–G)** The expression of four mRNAs from m^6^AIGs-PS in GC cells and GES-1. *p < 0.05, **p < 0.01.

To acquire candidate small molecules for treating GC, we uploaded the DEGs (|fold change| > 1 and *p* < 0.05) between different risk subgroups to the CMap database for GSEA. The top ten small molecules with satisfactory enrichment scores were listed in **Table 1** and the 3D chemical structures of the three most significant drugs were shown in **Figure 14A-C**, which may become new therapeutic regimens to treat GC.

**Table 1:** Results of CMap analysis.

Cmap name	Mean	*n*	Enrichment	*p*	Specificity	Percent non-null
ikarugamycin	-0.698	3	-0.981	0.00004	0	100
iproniazid	-0.584	5	-0.904	0.00004	0	100
fludrocortisone	-0.319	8	-0.635	0.0013	0.0704	50
etiocholanolone	-0.327	6	-0.698	0.00181	0.0714	50
eticlopride	-0.422	4	-0.817	0.00205	0	75
midodrine	-0.362	5	-0.739	0.00246	0.0526	60
lasalocid	-0.328	4	-0.808	0.00263	0.0278	50
gentamicin	-0.486	4	-0.808	0.00265	0.0461	75
amitriptyline	-0.17	6	-0.673	0.00328	0	50
naringenin	-0.565	4	-0.785	0.00426	0.0323	75

### Validation of the expression levels of the prognostic m6AIGs in cell lines

Human GC cell line MGC-803, BGC and AGS, and normal human gastric epithelial cell line GES-1 were used to validate the expression levels of the m^6^AIGs from m^6^AIGs-PS. As a result, PAEP and FANCC were upregulated in GC cells compared with GES-1, and GLP2R was lowly expressed in GC cells, which were with the same expression profile with TCGA data (**Figure 14D-F**). However, we found that NPR1 was upregulated in GC cells compared with GES-1, which was not consistent with the outcome in the TCGA cohort. (**Figure 14G**).

## Discussion

Emerging data have suggested that both the global m^6^A levels and the expression of m^6^A regulators are dysregulated in various types of cancers, which are associated with drug resistance and prognosis (42). Additionally, copious studies have shown that m^6^A take a key part in immune recognition and activation of immune responses (26, 43). Thus, we comprehensively evaluated the prognostic roles and the function of m^6^AIGs and revealed the latent relationship between m^6^AIGs with TME in GC.

In previous investigations, the immune-related genes or m^6^A-related regulators signatures for prognostic prediction have been studied in a variety of cancers, such as lung Adenocarcinoma (44, 45), hepatocellular carcinoma (46, 47), etc. Similarly, based on the differentially expressed immune-related genes or m^6^A-related regulators, several prognostic signatures have also been established to predict the outcome of GC patients. A three m^6^A-related regulators signature (FTO, RBM15, ALKBH5) could be used to effectively predict the clinicopathological features of GC patients (48), and a nine m^6^A-related lncRNA risk signature is an independent prognostic indicator for patients with GC, which can effectively predict survival status (49). Another ten immune-related genes signature has been constructed to evaluate the immune landscape of GC patients and found related regulatory mechanisms in GC (50). However, the immune-related genes–m^6^A-related regulators interaction in the GC prognostic model remains to be clarified. Here we report for the first time the m^6^AIGs signature for prognosis and immune landscape of GC populations. In addition, *via* a comprehensive bioinformatic method as previous studies (51, 52), we found different clusters based on the m^6^AIGs in GC, explored the risk of different clinicopathological features, TME scores, TIICs and ICGs, and acquired candidate small molecules for treating GC.

A total of 443 GC patients and 407 samples from the TCGA datasets, 23 m^6^A-related regulators and 3179 immune-related genes were included in our study. Fourteen m^6^AIGs were confirmed to have prognostic value, and GC patients were segmented into two clusters (clusters I and II) according to the expression of those m^6^AIGs. There were significant differences in OS rates, age, and tumor grade between these two clusters. Subsequently, four prognosis-related m^6^AIGs, namely PAEP, GLP2R, NPR1, and FANCC, were used to establish a prognostic signature for predicting the OS of GC patients. PAEP, a direct T cell inhibitor, seems to promote tumor growth by negatively regulating the anti-tumor immune response. For instance, the melanoma cell-secreted PAEP could suppress the activation and cytotoxicity of T lymphocytes, resulting in immune tolerance within the TME (53). There is little known about the relationship between GLP2R and cancer, but local activation of GLP2R diminishes islet inflammation by attenuating macrophage activation (54). NRP1 has been reported to be an oncogene by participating in the development and progression of cancers, such as lung adenocarcinoma (55), breast cancer (56), prostate cancer (57), and gastric cancer (58). Several studies have provided evidence that FANCC-deficient mononuclear phagocytes could produce multiple inflammatory cytokines (59), and FANCC disruption caused increased spontaneous chromosomal breakage, clastogenic damage on irradiation, and clastogenic damage (60). Significant differences in OS were observed between different risk subgroups under KM analyses and ROC curve analysis confirmed that m^6^AIGs-PS had a good predictive performance. Univariate Cox and multivariate Cox analyses demonstrated RS can be used as an independent prognostic factor for GC. The results indicated the specificity and sensitivity of m^6^AIGs-PS in GC. In addition, we established a nomogram comprising RS and clinical risk characteristics, which had particular importance in clinical practice. Furthermore, patients stratified by RS had different clinical characteristics, such as age, grade, stage M, and stage N. RS also could serve as a prognostic instrument for patients with diverse clinical features, especially age, sex, tumor grade G3, clinical stage, stages T3–T4, stage M0, and stage N.

TME has been identified as a key modulator of tumor progression and prognosis of cancer patients for more than a decade (61). ICGs are novel targets to develop treatments for tumors and have shown excellent clinical benefit, resulting from their roles in circumventing self-reactivity (62). Hence, the relationship between m^6^AIGs and the immune landscape including TME scores, TIICs, and ICGs expression was analyzed. The tumor purity decreased in high-risk groups and cluster II was associated with poorer OS, suggesting the malignant effect of stromal cells and the immune exhaustion in GC. In tumors, the coordinated intercellular interactions that exist in normal tissues are destroyed as the cancer cells obtain the capacity to chronically circumvent normalizing cues from the microenvironment (63). For example, a study using a mouse model of inflammation-associated GC demonstrated that cancer-associated fibroblasts promote GC cell growth and progression *via* secretion of CXCL12, Wnt5a, and IL-6 (9). Moreover, CXCL1 secreted by tumor-associated lymphatic endothelial cells promoted the migration, invasion, and adhesion of GC cells by upregulating MMP9, MMP2, and integrin β1 (64). Concerning TIICs, monocytes positively correlated with cluster II and the high-risk group. It has been well established that monocyte-derived tumor-associated macrophages could promote tumorigenesis by remodeling the extracellular matrix and immune suppression (65). Immune checkpoint inhibitors (ICIs) have revolutionized cancer treatment with excellent efficacy in different cancers (66). The analysis of ICGs showed that some prominent ICGs, such as CD28, CD86, CD40, TNFSF4, and TNFSF18, were significantly overexpressed in cluster II and the high-risk group.

Functional enrichment analysis was performed between different clusters or different risk subgroups to view the latent functions of m^6^AIGs. GO analysis results revealed that the DEGs in different risk subgroups and clusters were particularly enriched in some cancer-related biological processes, such as “extracellular matrix organization”, “collagen-containing”, and “extracellular matrix structural constituent” (67, 68). Collagen, the main ingredient of the extracellular matrix and the interstitial microenvironment, could offer a scaffold for tumor cells and cause tumor migration. Similarly, the results of KEGG pathways analysis also enriched in malignancy-associated pathways of GC, containing “PI3K-Akt signaling pathway”, and “Wnt signaling pathway”. The results of GSEA showed that cluster II and the high-risk group were enriched in some malignant pathways, such as “Focal adhesion”, “ECM−receptor interaction”, and “Cell adhesion molecules”.

Finally, some candidate drugs with the potential to reverse aberrant gene expression in GC were identified. The above-selected drugs are untraditional anti-tumor drugs, but there is lots of evidence of their effects on cancer cells. Ikarugamycin, isolated from mangrove-derived *S. xiamenensis* 318, could block glycolysis in pancreatic cancer by targeting HK2 (69) and induce apoptosis in HL-60 leukemia cells through genotoxicity (70). Therefore, the drugs with potential efficacy should be further studied and tried in the clinical trial.

Consequently, the m^6^AIGs-PS was helpful for the risk stratification of GC patients, and the nomogram had the latent force to be used as a quantitative instrument to predict OS. Besides, searching for effective therapeutic targets to improve the limitation of current immunotherapy for GC patients is urgent. In our research, we explored the immune-related genes–m6A-related regulators interaction, which provides a promising strategy with an important clinical implication for guiding individual therapy based on m^6^A RNA modification.

However, there are some limitations in our research. Firstly, our findings are based on the online database, and the predictive performance of m^6^AIGs-PS still needed to verify in the large-sample clinical cohort in the future. Secondly, our research mainly focused on bioinformatic analysis, more experimental studies exploring the specific role of m^6^AIGs in GC are in urgent need in future work.

## Conclusion

The prognostic value and significance in the assessment of the immune landscape of m^6^AIGs in GC patients were systematically analyzed in this study. Two clusters based on 14 prognostic m^6^AIGs were identified. A prognostic signature based on 4 m^6^AIGs and a nomogram based on independent prognostic factors was constructed and validated. These results had significant value in predicting the OS of GC patients, clinicopathological features, and TME. Some biological processes and pathways associated with cancer and immune response were enriched, which provides a basis for further exploring the function of m^6^AIGs. Lastly, some potential small molecule drugs for the therapy of GC were identified.

## Data availability statement

The original contributions presented in the study are included in the article/supplementary material. Further inquiries can be directed to the corresponding authors.

## Ethics Statement

This study met the publication guidelines stated by TCGA (https://cancergenome.nih.Gov/publications/publicationguidelines). Ethics approval and informed consent were not required.

## Author contributions

YH, YW, JL, and FL designed the study. YH, YZ, YT, and ZY organized the database, performed statistical analyses, and prepared the figures and tables. YH, YZ, and YW wrote the first draft of the manuscript. ZH, PL, FL, JL, and YW revised the manuscript. All authors contributed to the article and approved the submitted version.

## Funding

This study was supported by The National Natural Science Foundation (No. 81904139, No. 81973819, No. 81904145, No. 82104602, and No. 81903963), Collaborative Innovation Team Project of First-Rate Universities and Disciplines and High-level University Discipline of Guangzhou University of Chinese Medicine (No. 2021xk47), The Natural Science Foundation of Guangdong Province (No. 2019A1515011145 and No. 2018A030310614), Guangdong Medical Science and Technology Research Fund (No. B2021089, No. A2020186), Guangzhou Science and Technology Project (No. 202102020535), Clinical Research Project of Innovation Hospital in the First Affiliated Hospital of Guangzhou University of Chinese Medicine (No. 2019IIT19) and Innovation Development project of the First Affiliated Hospital of Guangzhou University of Chinese Medicine (No. 2019QN01).

## Acknowledgments

We appreciate TCGA database and the authors up-loaded their data. This work is supported by the Lingnan Medical Research Center of Guangzhou University of Chinese Medicine. We are grateful to acknowledge Dr. Hong Mi (Department of Gastroenterology, the First Affiliated Hospital of Guangzhou University of Chinese Medicine) for her valuable suggestions in revision.

## Conflict of interest

The authors declare that the research was conducted in the absence of any commercial or financial relationships that could be construed as a potential conflict of interest.

## Publisher’s note

All claims expressed in this article are solely those of the authors and do not necessarily represent those of their affiliated organizations, or those of the publisher, the editors and the reviewers. Any product that may be evaluated in this article, or claim that may be made by its manufacturer, is not guaranteed or endorsed by the publisher.
